# Efficient Delivery of CRISPR-Cas9 RNP Complexes with Cyclodextrin-Based Nanosponges for Enhanced Genome Editing: TILD-CRISPR Integration

**DOI:** 10.3390/ijms262110682

**Published:** 2025-11-02

**Authors:** Shahin Amiri, Setare Adibzadeh, Yousef Khazaei Monfared, Saeed Kaboli, Arash Arashkia, Farzaneh Barkhordari, Mohammad Mahmoudian, Mohammad Hassan Kheirandish, Francesco Trotta, Fatemeh Davami

**Affiliations:** 1 Department of Medical Biotechnology, Biotechnology Research Center, Pasteur Institute of Iran, Tehran 13169-43511, Iran; shaahinamiri@gmail.com (S.A.); 2 Student Research Committee, Pasteur Institute of Iran, Tehran 13169-43511, Iran; 3 Department of Nanoscale Science and Engineering, College of Nanotechnology, Science & Engineering University at Albany, State University of New York, Albany, NY 12222, USA; setareadibzade@gmail.com; 4 Division of Nuclear Medicine and Molecular Imaging, Department of Radiology, Massachusetts General Hospital, Harvard Medical School, Boston, MA 02114, USA; ykhazaeimonfared@mgh.harvard.edu; 5 Department of Chemistry, University of Torino, via P. Giuria 7, 10125 Torino, Italy; 6 Department of Medical Biotechnology, School of Medicine, Zanjan University of Medical Sciences, Zanjan 45137-66371, Iran; 7 Department of Molecular Virology, Pasteur Institute of Iran, Tehran 13169-43511, Iran; 8 Candiolo Cancer Institute, FPO-IRCCS, 10060 Candiolo, Italy; 9 Department of Medical Biotechnology, School of Medicine, Shahid Sadoughi University of Medical Sciences, Yazd 89151-73143, Iran

**Keywords:** CRISPR-Cas, CHO cells, genome editing, homology-directed repair, ribonucleoproteins, nanoparticles, nano delivery system

## Abstract

The CRISPR-Cas9 system has transformed biomedical research by enabling precise genetic modifications. However, efficient delivery of CRISPR components remains a major hurdle for therapeutic applications. To address this, we employed a new modified cationic hyper-branched cyclodextrin-based polymer (Ppoly) system to deliver an integrating GFP gene using the TILD-CRISPR method, which couples donor DNA linearization with RNP complexes. The physicochemical properties, loading efficiency, and cellular uptake of RNP with Ppoly were studied. After transfection, antibiotic selection and single-cell cloning were performed. Junction PCR was then performed on the isolated clones, and we compared the knock-in efficiency of Ppoly with that of the commercial CRISPRMAX™ reagent (Thermo Fisher, Invitrogen™, Waltham, MA, USA). The results demonstrate the encapsulation efficiency of over 90% for RNP and Ppoly, and cell viability remaining above 80%, reflecting the minimal toxicity of this approach. These attributes facilitated successful GFP gene integration using the TILD-CRISPR with RNP delivered via cyclodextrin-based nanosponges. The present method achieved a remarkable 50% integration efficiency in CHO-K1 cells, significantly outperforming the 14% observed with CRISPRMAX™ while maintaining lower cytotoxicity. This study highlights a promising platform for precise and efficient genome editing, with strong potential for therapeutic and regenerative medicine applications.

## 1. Introduction

Clustered regulatory interspaced short palindromic repeats (CRISPR)/CRISPR-associated protein 9 nuclease (Cas9), a third-generation genome editing tool, has emerged as a highly effective tool for precise genome editing, finding utility in various applications such as gene disruption, correction, regulation of transcription and translation, and cell line engineering across different systems [1]. The typical Type II CRISPR-Cas9 system employs a Cas9 enzyme and a single guide RNA (sgRNA) to target and cleave specific genomic sites, facilitating gene knockouts or insertion through genetic repair pathways. A distinct application of this technology lies in the targeted integration of genes to produce recombinant proteins, predominantly through the homology-directed repair (HDR) pathway, with the non-homologous end-joining (NHEJ) mechanism contributing to a lesser extent [2,3]. This approach can target transcriptionally active regions (safe harbors or hot spots) of the genome in animal cells, particularly Chinese hamster ovary (CHO) cells, which serve as the leading host for biotherapeutic production in mammalian expression systems, facilitating genetic engineering to enhance recombinant protein production [4,5]. Additionally, it ensures improved stability in both quality and gene expression across successive cell passages over time. In contrast, conventional methods based on random integration (RI) often lead to clonal instability and decreased productivity, ultimately reducing the overall efficiency of these clones [6]. Given that the HDR rate in mammalian cells is inherently lower than that of NHEJ, numerous strategies have been developed in recent years to significantly enhance the occurrence of HDR events for diverse applications in cell engineering and disease treatment [7]. These approaches range from biotechnological modifications of donor templates and CRISPR components to alterations in cell culture conditions and advancements in delivery strategies. Such innovations aim to improve delivery efficiency, reduce cellular mortality rate, and ultimately enhance the integration and expression of target proteins [8].

Recent advancements have explored various strategies to enhance HDR efficiency, with donor template design playing a central role. Single-stranded DNA oligonucleotides (ssODNs) have been widely used to induce point mutations and for gene tagging [9,10,11], but their size limitations have shifted the focus toward long single-stranded DNA (lssDNA) templates [12,13]. Methods such as Easi-CRISPR [14] and CLICK [15] have emerged as solutions, yet their efficiency declines when donor lengths exceed 2 kb. To address these limitations, researchers have explored double stranded DNA (dsDNA) donors, which offer greater flexibility in sequence design and integration efficiency. However, challenges such as random integration (RI) and lower HDR success rates persist when using vector-based donors. Alternative techniques such as HITI [16] and PITCh [17] have been developed to improve HDR outcomes, with PITCh utilizing microhomology sequences and multiple sgRNAs to facilitate precise transgene integration. While these approaches enhance efficiency [5,18,19], they remain constrained by donor preparation methods and the complexity of integration mechanisms. As a result, recent research has focused on optimizing dsDNA donor linearization via polymerase chain reaction (PCR) amplifications or restriction enzymes, a strategy exemplified by the targeted integration with linearized dsDNA (TILD)-CRISPR method [20,21]. This approach facilitates precise integration of exogenous DNA sequences into the genome at predefined loci by enhancing HDR efficiency through the linearization of donor DNA. This method has significantly enhanced the efficiency of HDR in mouse embryos, the brain tissue of mice, and human embryos. Furthermore, it has been successfully utilized for precise transgene integration in human T cells [22].

Pre-assembled Cas9 protein and in vitro-synthesized sgRNA (Ribonucleoprotein (RNP) complexes) enable rapid and transient genome editing, as they bypass transcription and translation steps required by mRNA or plasmid systems and degrade quickly after delivery, enhancing specificity while minimizing prolonged residual editing activity and off-target mutations [23]. Unlike plasmid-based systems, RNPs eliminate the risk of unintended integration of foreign DNA into the host genome, enhancing safety [24,25]. This approach is also precise, robust, capable of multiplexing, and less likely to trigger immune responses compared to plasmid- or mRNA-based CRISPR systems, making them suitable for therapeutic applications [26].

Despite the remarkable potential of the CRISPR-Cas9 tool, the efficient intracellular delivery of this substantial RNP complex remains a major bottleneck for clinical application. Ensuring serum stability and reducing cytotoxicity, off-target mutations, and immunogenic responses are crucial for advancing the delivery technology. Various mechanisms have been used to deliver the RNP complexes, including electroporation, microinjection [27,28], extracellular vesicles, and viral carriers [29]. But non-viral delivery systems—such as inorganic nanoparticles (NPs), liposomes, and cationic polymeric NPs—have garnered considerable attention in the scientific community due to their scalability, reduced immunogenicity, and capacity to carry large DNA plasmids and RNA molecules [30,31]. However, these systems are often limited by their relatively low transfection efficiency. Among the wide range of nanocarriers explored, cyclodextrins (CDs) have emerged as an up-and-coming and effective platform for drug delivery, especially in the context of oligonucleotide-based therapies [32,33,34]. These nanocarriers address several limitations inherent to other systems, such as poor solubility and rapid degradation, by offering enhanced stability, superior loading capacity, and controlled therapeutic agent release [35,36]. CDs are cyclic oligosaccharides composed of glucopyranoside units connected by α-(1,4)-glycosidic bonds, forming a structure with a hydrophobic central cavity and a hydrophilic exterior. Their unique architecture has sparked growing interest in their use across the pharmaceutical, nutraceutical, and related industries. Notably, natural CDs are classified as “Generally Recognized as Safe” (GRAS), further supporting their use in biomedical applications [37,38,39]. These innovative structures have demonstrated remarkable advantages in the delivery of drugs and other biological macromolecules [40]. To enhance their interaction with oligonucleotides, cationic CD derivatives and CD-based polymers containing cationic functional groups have been developed [41].

**Figure 1:**
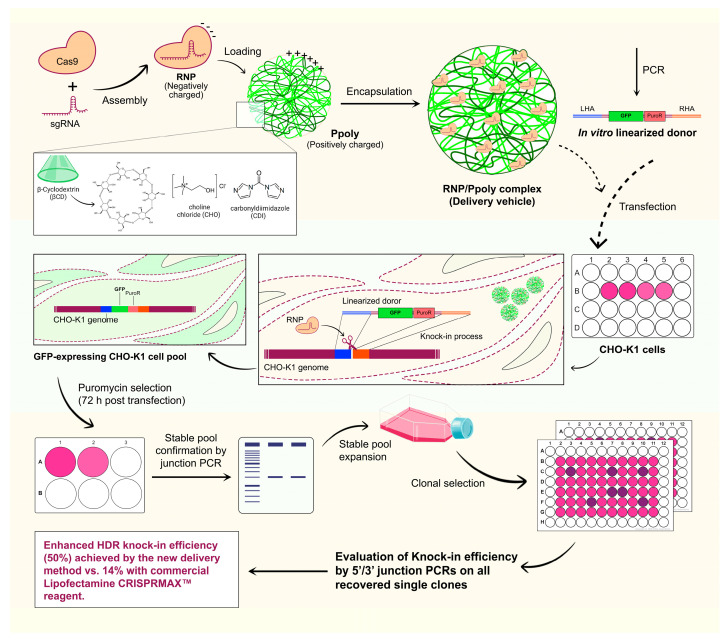
Schematic Overview of the Targeted Integration with Linearized dsDNA (TILD)-CRISPR Knock-In (KI) Strategy Using the Cationic Hyper-Branched Cyclodextrin-Based Polymer (Ppoly). The Illustration of the TILD-CRISPR platform, in which Ppoly serve as delivery vehicles for the stable and targeted transport of CRISPR–Cas9 RNP complexes for efficient intracellular delivery. The RNP/Ppoly complexes, together with linearized donor DNA, are transfected into Chinese hamster ovary (CHO)-K1 cells to promote homology-directed repair (HDR)-mediated KI. Green fluorescent protein (GFP)-expressing stable pools are subsequently selected and validated by junction polymerase chain reaction (PCR), demonstrating the effectiveness of this delivery strategy for precise genome editing in mammalian cells.

In this regard, cationic hyperbranched cyclodextrin-based polymers (Ppoly) have been synthesized using choline chloride to introduce positive charges and carbonyldiimidazole (CDI) as a crosslinking agent, enabling their use as effective delivery vehicles—specifically for transporting CRISPR components in the structure of RNP complexes. Instead of utilizing plasmids expressing Cas9 and sgRNA or mRNA encoding Cas9, we opted for purified Cas9 protein and in vitro-synthesized sgRNA to further enhance integration efficiency. The nanosponge architecture not only improves stability and biocompatibility but also facilitates the effective transport of RNP complexes to target cells. Moreover, in this study, we replaced donor plasmids with in vitro linearized dsDNA donors featuring 1000-base-pair homology arms. Integration efficiency and specificity were meticulously evaluated through molecular techniques and fluorescence microscopy. This research highlights the promise of combining TILD-CRISPR, RNP complexes, and Ppoly as a robust delivery platform to boost Knock-in (KI) efficiency and enable precise genome editing, with significant potential for cell line engineering and therapeutic applications, including gene therapy and regenerative medicine. This adaptable system offers extensive opportunities across various Nanobiotechnology and Medicine domains. The overall strategy for CRISPR-Cas9 RNP delivery is schematically illustrated in Figure 1.

## 2. Results

### 2.1. Preparation and Characterization of RNP/Ppoly Complexes

#### 2.1.1. Fourier Transform Infrared (FTIR) Analysis

**Figure 2:**
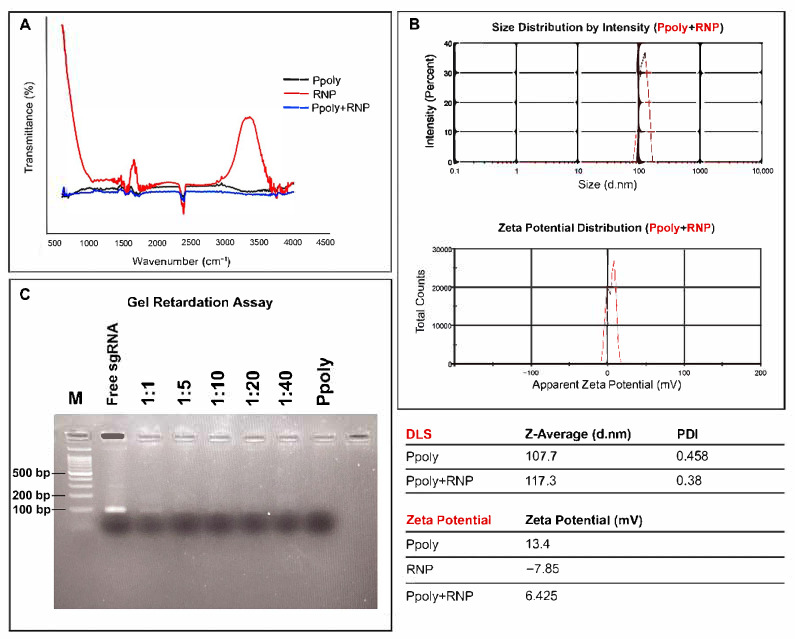
(**A**) Fourier transform infrared (FTIR) Spectra of cationic hyper-branched cyclodextrin-based polymers (Ppolys), Ribonucleoprotein (RNP), and RNP-loaded Ppoly: FTIR spectra of Ppolys, RNP, and the RNP/Ppoly complex over the wavelength range of 500 to 4500 cm^−1^. The RNP spectrum displays significant peaks around 1500 cm^−1^ and 3500 cm^−1^, indicating prominent molecular interactions. (**B**) Dynamic light scattering (DLS) and Zeta Potential Analysis: Particle size distribution of RNP/Ppoly complexes with an average size of 117.3 nm, indicating successful encapsulation of RNP. Zeta potential distribution for RNP/Ppoly complexes with a peak at +6.425 mV, showing partial neutralization of the negative charge of RNP by positively charged Ppoly. (**C**) Gel retardation assay of Ppoly/single guide RNA (sgRNA) complexes at various N/P ratios. Lane M: molecular weight marker; Lane sgRNA: free sgRNA; Lanes 1:1, 1:5, 1:10, 1:20, and 1:40: Ppoly/sgRNA complexes at corresponding N/P ratios; Lane Ppoly: polymer only. At all tested ratios, sgRNA migration was inhibited, indicating a strong interaction between Ppoly and sgRNA.

The spectra of Ppoly, RNP, and RNP-loaded Ppoly are compared over the wavelength range of 500 to 4500 cm^−1^ (Figure 2A). The RNP spectrum (red line), representing the Cas9–sgRNA RNP complex, displays a broad peak around 3300–3400 cm^−1^, attributed to O–H and N–H stretching vibrations, which are characteristic of both RNA and protein components. Notably, strong absorbance bands appear at approximately 1650 cm^−1^ and 1540 cm^−1^, corresponding to the amide I (C=O stretching) and amide II (N–H bending + C–N stretching) bands, respectively. These amide bands arise from the peptide backbone of the Cas9 protein, confirming the proteinaceous nature of the complex. Additional minor peaks observed in the 1000–1250 cm^−1^ range are likely associated with phosphate group vibrations from the RNA backbone. In contrast, the Ppoly spectrum (black line) shows a relatively flat baseline with weak signals in these regions, reflecting the synthetic polymer’s distinct chemical structure. Upon complexation with RNP (blue line), the spectrum of the Ppoly/RNP complex exhibits broadening and dampening of the O–H/N–H stretch, as well as reduced intensity in the amide I and II regions, suggesting intermolecular interactions. These spectral changes are indicative of electrostatic interactions and hydrogen bonding between the cationic amine groups of Ppoly and the polar/charged areas of the RNP complex, supporting successful complex formation.

#### 2.1.2. Dynamic Light Scattering (DLS) and Zeta Potential Analysis

The DLS and zeta potential measurements were conducted to assess the size and surface charge of Ppolys and their complexes with RNP. The results, shown in Figure 2B, provide valuable insights into the encapsulation efficiency and stability of the nanocarrier system. Particle Size Analysis: The size distribution of Ppoly and RNP/Ppoly complexes was determined using DLS. The average size of Ppoly alone was approximately 107.7 nm, while the RNP/Ppoly complex exhibited an average size of 117.3 nm. The increase in particle size upon RNP encapsulation indicates successful complex formation. Zeta Potential Analysis: The zeta potential measurements of the RNP/Ppoly complex are shown in Figure 2B, respectively. The zeta potential of Ppoly alone was found to be approximately +13.4 mV, indicating a positively charged surface. The zeta potential of RNP alone was approximately −7.86 mV, reflecting its negative charge. Upon complexation with Ppoly, the RNP/Ppoly complex exhibited a zeta potential of roughly +6.425 mV, indicating partial neutralization of the negative charge of RNP by the positively charged Ppoly. These results demonstrate that the Ppoly surface is positively charged, enabling effective interaction with the negatively charged RNP. The resulting RNP/Ppoly complex maintains a positive overall charge, which is essential for stable encapsulation and efficient delivery. In summary, the DLS and zeta potential analyses confirm the formation of stable RNP/Ppoly complexes and suggest favorable physicochemical properties for gene delivery applications, although further studies are needed to determine the precise mode of encapsulation.

#### 2.1.3. Qualitative Analysis of Gel Retardation

The gel retardation assay was performed to qualitatively assess whether the sgRNA forms stable complexes with Ppoly at different N/P ratios. As shown in Figure 2C, at all examined concentrations, sgRNA was well encapsulated and did not migrate through the gel. The lanes labeled “M,” “sgRNA,” “1:1”, “1:5”, “1:10”, “1:20”, “1:40”, and “Ppoly” represent various ratios of Ppoly to sgRNA, with “M” being the molecular weight marker. The lack of movement in the gel indicates that the sgRNA forms stable complexes with Ppoly, preventing its migration. This result demonstrates the efficient encapsulation of sgRNA by Ppoly at different ratios, highlighting the potential of these nanocarriers for effective gene delivery systems.

### 2.2. 3-(4,5-Dimethylthiazol-2-yl)-2,5-diphenyltetrazolium Bromide (MTT) Assay

**Figure 3:**
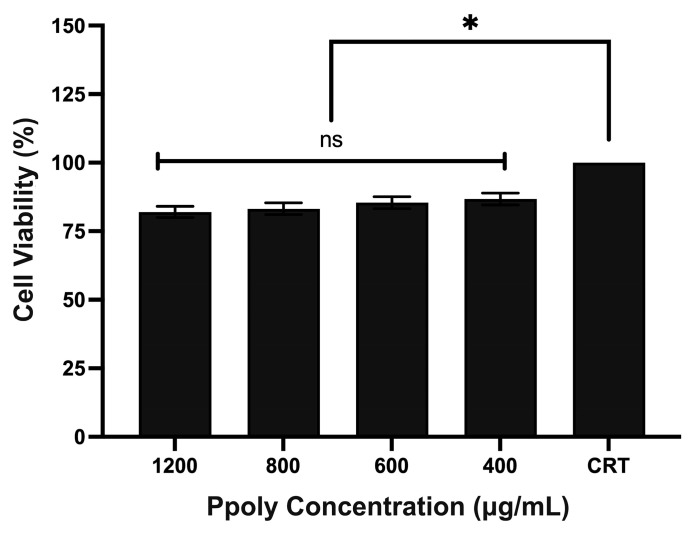
Evaluation of Cytotoxicity Using the 3-(4,5-Dimethylthiazol-2-yl)-2,5-Diphenyltetrazolium Bromide (MTT) Assay: Bar graph depicting the cell viability (%) of Chinese hamster ovary (CHO)-K1 cells treated with cationic hyper-branched cyclodextrin-based polymers (Ppolys) at various concentrations (400 µg/mL, 600 µg/mL, 800 µg/mL, 1200 µg/mL) compared to untreated control cells. The results demonstrate a slight, concentration-dependent decrease in cell viability. Despite this, even at the highest concentration (1200 µg/mL), cellular viability remains above 80%, indicating the relatively low cytotoxicity of Ppolys. These findings support the potential use of Ppolys as effective nanocarriers with minimal adverse effects on cell viability. * *p* < 0.05 and ns (not significant).

The cytotoxicity of the Ppolys was evaluated using the MTT assay at various concentrations. CHO-K1 cells were cultured and treated with different concentrations of the nanosponges for 24 h. The results are shown in Figure 3. The cell viability of CHO-K1 cells to varying concentrations of the cyclodextrin nanosponges is as follows: 1200 µg/mL: 82.07%; 800 µg/mL: 83.24%; 600 µg/mL: 85.44%; 400 µg/mL: 86.74%; Control (untreated): 100%. Although not statistically significant, the lower concentrations of cyclodextrin nanosponges (400 µg/mL and 600 µg/mL) showed less cytotoxicity compared to higher concentrations (800 µg/mL and 1200 µg/mL). Remarkably, even at higher concentrations (800 µg/mL and 1200 µg/mL), the cellular viability remained above 80%, indicating that the Ppolys exhibit relatively low cytotoxicity. This demonstrates the potential of these nanocarriers for efficient delivery with minimal adverse effects on cell viability.

### 2.3. Cellular Uptake and Encapsulation Efficiency of RNP/Ppoly

**Figure 4:**
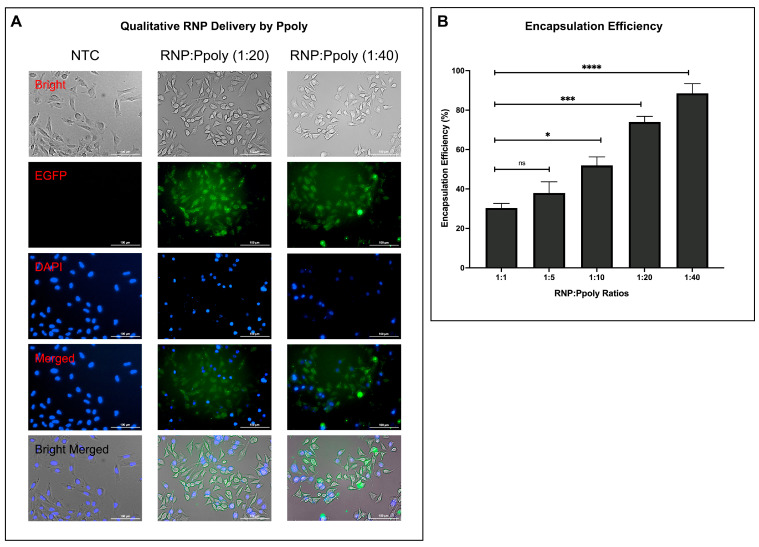
Comprehensive Analysis of Ribonucleoprotein (RNP) Delivery and Encapsulation Efficiency: (**A**) RNP Delivery Efficiency: A qualitative analysis showing RNP (composed of single guide RNA (sgRNA) and Cas9-GFP-NLS) entry into cells at two concentrations: 1:20 and 1:40. The leftmost column represents non-transfected control (NTC) cells, which have not undergone any modification and serve as a baseline control. The images include: Bright-field (Bright): Cell morphology and structure. Enhanced green fluorescent protein (EGFP) fluorescence: Green fluorescence indicates RNP localization and entry. 4′,6-diamidino-2-phenylindole (DAPI) fluorescence: Blue fluorescence highlights nuclei for cellular referencing. Merged fluorescence (Merged): Co-localization of RNP and nuclei. Bright-field and fluorescence merged (Bright Merged): An integrated view of cell structure and RNP entry. Scale bar = 100 μm. (**B**) Encapsulation Efficiency: Depicts increasing RNP encapsulation with higher Ppoly ratios. The bar graph depicts the encapsulation efficiency of RNP at increasing Ppoly ratios up to 1:40, showing higher encapsulation efficiency at 1:40 than at lower ratios. * *p* < 0.05, *** *p* < 0.001 and **** *p* < 0.0001, and ns (not significant).

The qualitative analysis of RNP (comprising sgRNA and Cas9-GFP-NLS) entry into cells is presented in Figure 4A. The results are shown for two different N/P ratios: 1:20 and 1:40. In the left column, the result for the 1:20 ratio is displayed, while the right column shows the result for the 1:40 ratio. These images demonstrate the efficiency and localization of RNP entry into cells at different ratios, which is crucial for understanding the delivery and activity of the RNP complex in gene-editing experiments. Qualitatively, cells transfected at the 1:40 ratio appeared to show a stronger and more uniform Enhanced green fluorescent protein (EGFP) signal compared to the 1:20 condition, suggesting potentially more efficient uptake under these conditions.

The encapsulation efficiency of RNP with Ppoly was evaluated at different RNP:Ppoly ratios. The results, depicted in Figure 4B, demonstrate that as the concentration of the nanocarrier increases, the encapsulation efficiency of RNP also increases. These findings indicate that higher concentrations of Ppoly lead to better RNP encapsulation. Given the high solubility and low cytotoxicity of Ppoly observed in CHO-K1 cells at concentrations up to 1200 µg/mL, the N/P ratio of 1:40—corresponding to a working polymer concentration of approximately 800 µg/mL—was selected for further studies, as it falls within the biocompatible range. The increased encapsulation efficiency at higher concentrations suggests that Ppoly is an effective nanocarrier for RNP delivery, optimizing gene editing experiments by ensuring a high level of encapsulation.

### 2.4. 5′/3′ Junction PCR on Stable GFP-Expressing Cell Pool

**Figure 5:**
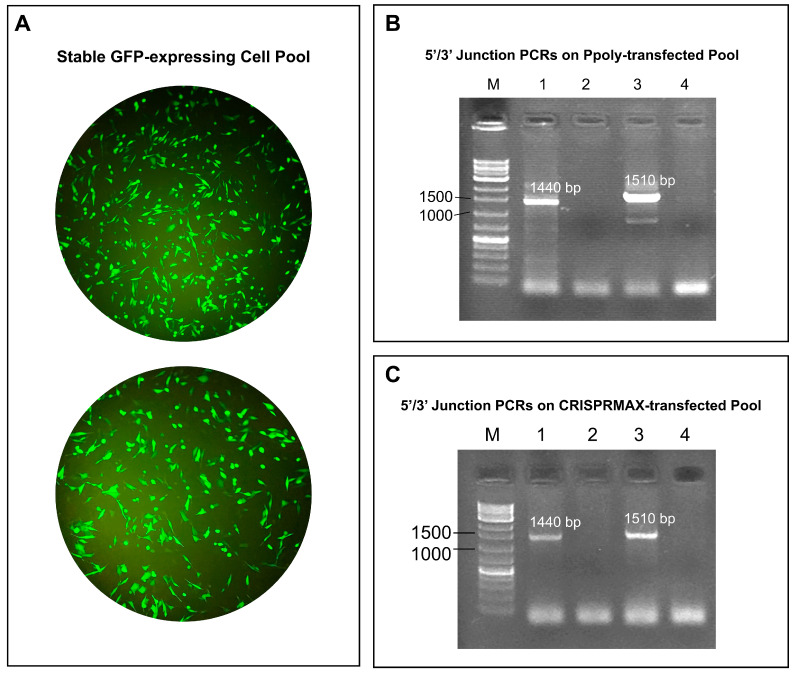
Validation of Stable Green Fluorescent Protein (GFP) Expression and Gene Delivery Efficiency: (**A**) Bright-field and fluorescence microscopy images of the stable GFP-expressing cell pool, displaying green fluorescence from GFP, confirm successful gene expression. (**B**,**C**) Gel electrophoresis analysis of the 5′/3′ junction polymerase chain reactions (PCRs) performed on cell pools transfected with cationic hyper-branched cyclodextrin-based polymer (Ppoly) (**B**) and CRISPRMAX (**C**). Lanes are labeled as follows: M: 1 Kb molecular weight marker. 1: DNA band corresponding to the expected size for the 5′ junction (1440 bp). 2: Negative control for the 5′ junction PCR. 3: DNA band corresponding to the expected size for the 3′ junction (1510 bp). 4: Negative control for the 3′ junction PCR. These findings validate successful PCR amplification, correct band sizes, and the stable integration of the GFP gene into the cell pool. The consistent band patterns across the experimental lanes and absence of bands in the negative controls highlight the reliability and effectiveness of both Ppoly and CRISPRMAX as gene delivery methods.

The results from the 5′/3′ Junction PCR on the cell pool with stable GFP expression, using both Ppoly carrier and CRISPRMAX reagent, are shown in Figure 5B,C. The gel electrophoresis image displays DNA bands in five lanes labeled M, 1, 2, 3, and 4. The bands in these lanes correspond to the expected sizes for the 5′ junction (1440 bp) and the 3′ junction (1510 bp), indicating successful PCR amplification and correct band sizes. These results demonstrate the accurate amplification of the target regions, confirming the stable integration and expression of the GFP gene in the cell pool. The consistent band sizes across different lanes further validate the reliability and efficiency of both Ppoly and CRISPRMAX methods for gene delivery and stable expression in the cells.

The stable GFP-expressing cell pool was harvested 11 days after transfection, following 6 days of antibiotic selection applied every other day. This timeline ensured the elimination of transient expression and the enrichment of cells with stable genomic integration prior to clonal expansion.

### 2.5. Comparative KI Efficiency of Ppolys and Lipofectamine CRISPRMAX^TM^ Delivery Systems

**Figure 6:**
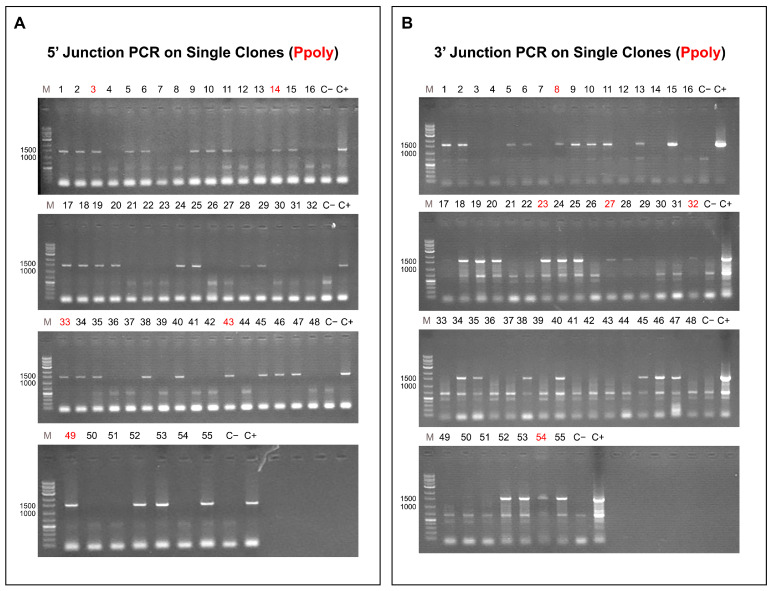
Analysis of Gene Insertion Efficiency Using the Cationic Hyper-Branched Cyclodextrin-Based Polymer (Ppoly) Carrier Method: (**A**) 5′ Junction polymerase chain reaction (PCR) Results: Gel electrophoresis results showing DNA bands for 55 single clones. Positive bands at 1440 bp were observed for clones 3, 14, 33, 43, and 49; however, these clones were excluded from calculations due to the absence of corresponding bands on the 3′ end. (**B**) 3′ Junction PCR Results: Gel electrophoresis results displaying DNA bands at 1510 bp for clones 8, 23, 27, 32, and 54. These clones were excluded from analysis as they lacked positive bands on the 5′ end. Insertion Efficiency: Among the 55 clones, 27 clones (1, 2, 5, 6, 9, 10, 11, 13, 15, 17, 18, 19, 20, 24, 25, 28, 29, 34, 35, 38, 40, 45, 46, 47, 52, 53, and 55) displayed positive bands on both the 5′ (1440 bp) and 3′ (1510 bp) junctions, resulting in an insertion efficiency of 49%. These results underscore the effectiveness of the Ppoly carrier method for achieving successful gene integration, with a substantial proportion of clones demonstrating correct, stable integration at both ends.

A: Results using Ppoly carrier: After clonal selection, 55 single clones were obtained. Each clone underwent 3′ junction PCR and 5′ junction PCR using cell lysate. Clones with positive bands on both ends were considered successful inserts for calculating insertion efficiency. 5′ Junction PCR Results: Figure 6A present the results of 5′ junction PCR for the Ppoly carrier method. Clones 3, 14, 33, 43, and 49 had positive bands (1440 bp), but they were excluded from calculations due to the lack of bands on the 3′ end. 3′ Junction PCR Results: Figure 6B show the results of 3′ junction PCR for the Ppoly carrier method. Clones 8, 23, 27, 32, and 54 had positive bands (1510 bp), but since these clones lacked bands on the 5′ end, they were also excluded from further analysis. Overall, 27 out of 55 clones (1, 2, 5, 6, 9, 10, 11, 13, 15, 17, 18, 19, 20, 24, 25, 28, 29, 34, 35, 38, 40, 45, 46, 47, 52, 53, and 55) had correct and positive bands on both 5′ and 3′ ends, showing an insertion efficiency of 49%. This indicates a relatively high success rate for gene insertion using the Ppoly carrier method. These results demonstrate the effectiveness of the Ppoly carrier method for achieving successful gene insertion, with a significant number of clones showing positive results on both ends of the target gene integration site.

**Figure 7:**
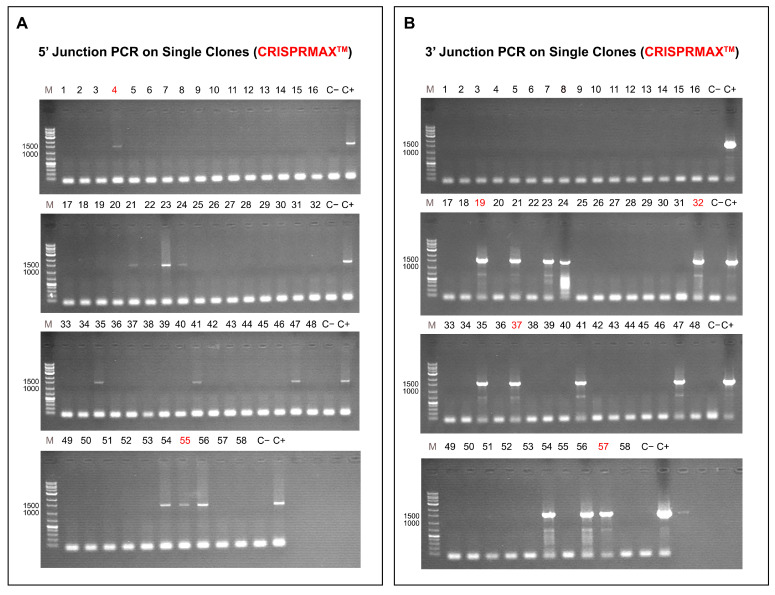
Gene Insertion Efficiency Analysis Using the CRISPRMAX Method: (**A**) 5′ Junction polymerase chain reaction (PCR) Results: Gel electrophoresis results showing DNA bands for 58 single clones. Clones 4 and 55 exhibited positive bands at 1440 bp but were excluded from analysis due to the absence of bands on the 3′ end. (**B**) 3′ Junction PCR Results: Gel electrophoresis results displaying DNA bands at 1510 bp for clones 19, 32, 37, and 57. These clones were excluded from further analysis as they lacked positive bands on the 5′ end. Insertion Efficiency: Out of 58 clones, 8 clones (21, 23, 24, 35, 41, 47, 54, and 56) displayed positive bands on both the 5′ (1440 bp) and 3′ (1510 bp) junctions, resulting in an insertion efficiency of 13.7%. These results indicate a lower success rate compared to the cationic hyper-branched cyclodextrin-based polymer (Ppoly) carrier method, emphasizing the significance of selecting an appropriate delivery system to optimize gene editing experiments.

B: Results Using CRISPRMAX: 58 single clones were obtained after clonal selection. Each clone underwent 3′ junction PCR and 5′ junction PCR using cell lysate. Clones with positive bands on both ends were considered successful inserts for calculating insertion efficiency. 5′ Junction PCR Results: Figure 7A presents the 5′ junction PCR results for the CRISPRMAX method. Clones 4 and 55 had positive bands (1440 bp), but they were excluded from calculations due to the lack of bands on the 3′ end. 3′ Junction PCR Results: Figure 7B shows the results of 3′ junction PCR for the CRISPRMAX method. Clones 19, 32, 37, and 57 had positive bands (1510 bp), but since these clones lacked bands at the 5′ end, they were also excluded from further analysis. Thus, in the CRISPRMAX method, 8 out of 58 clones (21, 23, 24, 35, 41, 47, 54, and 56) had correct and positive bands on both 5′ and 3′ ends, showing an insertion efficiency of 13.7%. This indicates a lower success rate compared to the Ppoly carrier method, highlighting the importance of choosing the appropriate delivery system for gene editing experiments.

### 2.6. Out-Out PCR Results

**Figure 8:**
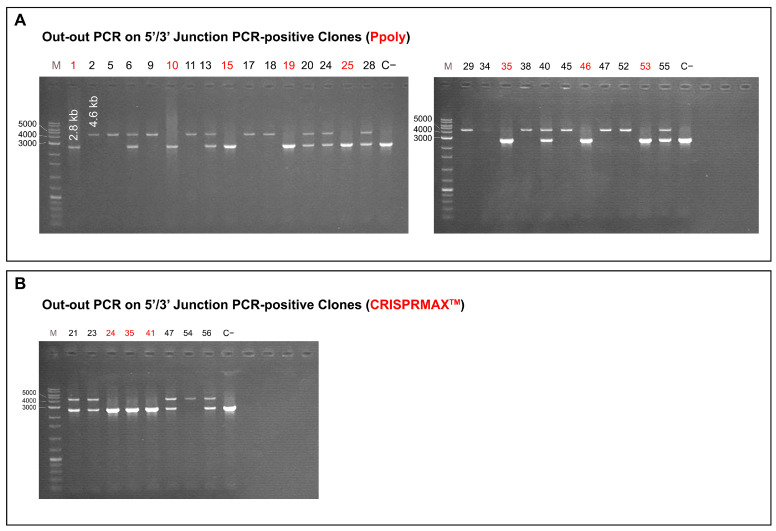
Out-Out Polymerase Chain Reaction (PCR) Results for Gene Construct Integration: (**A**) Ppoly Method: Gel electrophoresis results for out-out PCR analysis of 27 double-positive clones from the Ppoly nanocarrier method. Lanes show: Clones in red (1, 10, 15, 19, 25, 35, 46, 53): A single 2.8 kb band, indicating incomplete integration of the construct. Clones 6, 13, 20, 24, 28, 40, 55: Two bands (4.6 kb and 2.8 kb), indicating single-copy heterologous insertion. Clones 2, 5, 9, 11, 17, 18, 29, 34, 38, 45, 47, 52: A single 4.6 kb band, indicating double-copy homologous insertion. Of 27 clones, 19 (70%) were positive in out-out PCR, indicating higher efficiency for this method. (**B**) CRISPRMAX Method: Gel electrophoresis results for out-out PCR analysis of 8 double-positive clones. Lanes show: Clones in red (24, 35, 41): A single 2.8 kb band, indicating incomplete integration of the construct. Clones 21, 23, 47, 56: Two bands (4.6 kb and 2.8 kb), indicating single-copy heterologous insertion. Clone 54: A single 4.6 kb band, indicating double-copy homologous insertion. Of 8 clones, 5 (62%) were positive for out-out PCR, showing lower efficiency compared to the cationic hyper-branched cyclodextrin-based polymer (Ppoly) method. These findings highlight the superiority of the Ppoly method for achieving accurate, efficient gene integration, with a significantly higher proportion of clones showing complete, correct integration compared to the CRISPRMAX method.

The intact and complete integration of the gene construct was evaluated using out-out PCR analysis for each double-positive single clone from both delivery methods. Primers that anneal to the genomic locus outside the homologous arms were used. The results in each group showed the predicted band of approximately 4.6 kb. Heterologous insertion in clones resulted in two bands (4.6 kb and 2.8 kb), while homologous insertion produced a single 4.6 kb band. Figure 8A shows the out-out PCR results for targeted insertion using the Ppoly nanocarrier method. Clones indicated in red had a single 2.8 kb band, indicating amplification of the CHO-K1 genome at the locus without amplification of the inserted transgene. This suggests incomplete integration of the construct, which can occur due to various molecular reasons within the cell. Clones 6, 13, 20, 24, 28, 40, and 55, which had two bands (4.6 kb and 2.8 kb), indicate single-copy heterologous insertion of the transgene. Clones 2, 5, 9, 11, 17, 18, 29, 34, 38, 45, 47, and 52, which showed a single 4.6 kb band, indicate double-copy homologous insertion. In the Ppoly method, 19 out of 27 double-positive clones (70%) were identified as positive for out-out PCR, while the remaining clones (indicated in red) were negative. Figure 8B shows the out-out PCR results for targeted insertion using the CRISPRMAX method. Clones indicated in red had a single 2.8 kb band, indicating amplification of the CHO-K1 genome at the locus without amplification of the inserted transgene. This suggests incomplete integration of the construct. Clones 21, 23, 47, and 56, which had two bands (4.6 kb and 2.8 kb), indicate single-copy heterologous insertion of the transgene. Clone 54, which showed a single 4.6 kb band, indicates double-copy homologous insertion. In the CRISPRMAX method, 5 out of 8 double-positive clones (62%) were identified as positive for out-out PCR, while the remaining clones (indicated in red) were negative.

These results demonstrate that while both Ppoly and CRISPRMAX achieved successful gene insertion, Ppoly achieved higher overall efficiency and accuracy, highlighting its potential advantages for more reliable gene integration.

To further validate the specificity of the KI events, we used a two-step validation strategy. First, 5′/3′ junction PCR confirmed that the sgRNA guided Cas9 to the correct genomic locus. Then, out-out PCR verified precise integration beyond the homology arms. Importantly, real-time PCR results were consistent with out-out PCR, supporting accurate copy number and on-target insertion. Together, these complementary methods provide strong evidence for specific, HDR-mediated integration, minimizing the likelihood of off-target effects.

### 2.7. Insertion Copies Number Analysis

**Figure 9:**
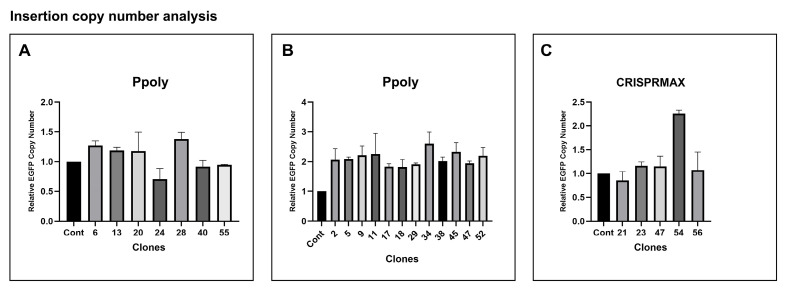
Insertion Copy Number Analysis Using Relative Real-time Polymerase Chain Reaction (PCR)**:** (**A**) Single-Copy Clones: Bar graph showing the relative green fluorescent protein (GFP) transgene copy number for out-out PCR-positive clones transfected using the cationic hyper-branched cyclodextrin-based polymer (Ppoly) nanocarrier method. Clones in this group have copy number values ranging between 0.5 and 1.5, indicating single-copy (heterologous) insertion. (**B**) Two-Copy Clones: Bar graph showing the relative GFP transgene copy number for out-out PCR-positive clones transfected using the Ppoly nanocarrier method. Clones in this group have copy number values ranging between 1.8 and 2.5, indicating double-copy (homologous) insertion in both alleles. (**C**) CRISPRMAX Method: Bar graph showing the relative GFP transgene copy number for out-out PCR-positive clones transfected using the CRISPRMAX reagent. Among these clones, only Clone 54 has a copy number value between 1.8 and 2.5, indicating double-copy (homologous) insertion. All other clones fall within the range of 0.5 to 1.5, indicating single-copy (heterologous) insertion. These results are entirely consistent with the out-out PCR findings, further validating the accuracy and efficiency of both methods in achieving successful gene integration, with the Ppoly method demonstrating a higher overall success rate.

To verify and confirm the copy number of inserted genes in clones that were positive for out-out PCR (heterologous or homologous), relative real-time PCR was performed. A clone with a single-copy GFP reporter gene (heterologously inserted into one allele) was used as a control, and other clones were compared to it. The 2^−ΔΔCt^ formula was used for calculations, with values ranging from 0.5 to 1.5 considered as single-copy clones, and values from 1.8 to 2.5 considered as double-copy (homologous insertion in both alleles). Figure 9 shows the relative GFP transgene copy number for single clones that tested positive in the Out-out PCR from each experimental group (transfected with Ppoly nanocarrier (Figure 9A,B) and CRISPRMAX reagent (Figure 9C)) using qRT-PCR. The error bars represent the standard deviation from two repetitions. These results provide a quantitative analysis of the copy number of inserted GFP genes in the selected clones, confirming the successful integration and expression of the transgene in the cell population. The relative real-time PCR results further validate the efficiency and reliability of both Ppoly and CRISPRMAX delivery systems in achieving accurate gene insertion.

### 2.8. Junction Validation and Genotyping

**Figure 10:**
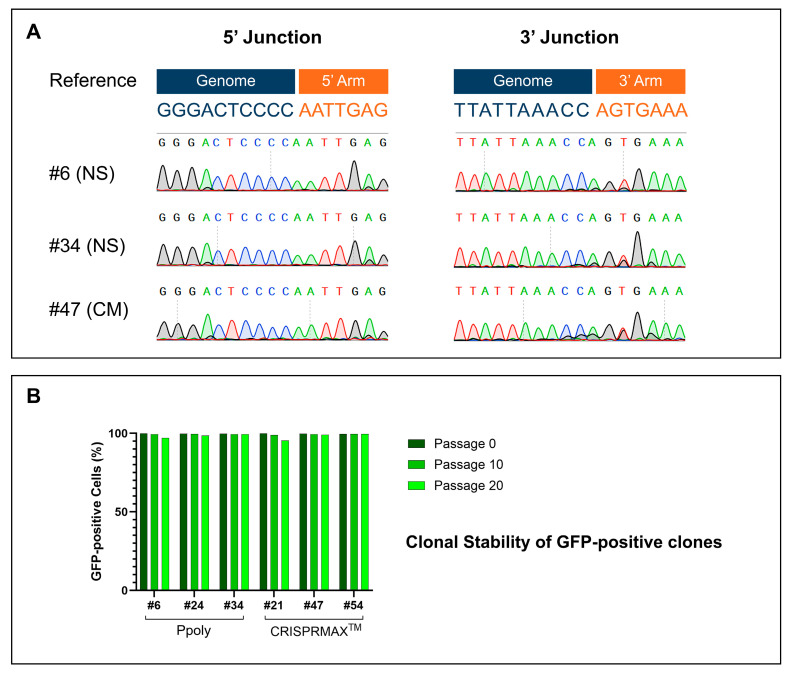
(**A**) Sanger Sequencing Results Showing the 5′ and 3′ Junctions of Green Fluorescent Protein (GFP)-Positive Clones: The reference sequences are provided at the top, with highlighted genome and arm sequences. Clones #6 and #34, derived through the cationic hyper-branched cyclodextrin-based polymer (Ppoly) method, are labeled as nanosponge (NS), while clone #47, obtained via the CRISPRMAX method, is labeled as CM. (**B**) Flow cytometry results assessing the clonal stability of GFP expression. GFP-positive clones were analyzed at passages 0, 10, and 20. Both Ppoly-derived (NS) and CRISPRMAX-derived (CM) clones maintained stable GFP expression levels over the 20-passage period.

To verify the fidelity of the 5′ and 3′ junctions, Sanger sequencing was conducted on three GFP-positive clones (Figure 10A). Among these, two clones (#6 and #34) were derived using the Ppoly method (denoted as nanosponge (NS) in the figure), and one clone (#47) was obtained using the CRISPRMAX method (represented as CM). Sequencing outcomes for both the 5′ and 3′ junctions revealed exceptional accuracy, as no insertions, deletions, or indels were detected, confirming the integrity of the junctions.

### 2.9. Stability Assessment of GFP-Positive Clones

The stability of GFP expression across generations was evaluated via flow cytometry. Three clones from each group (Ppoly and CRISPRMAX) were examined at passages 0, 10, and 20 (Figure 10B). The analysis demonstrated consistent GFP expression in all selected clones, indicating that GFP stability was preserved over time, even in the absence of puromycin selection pressure.

## 3. Discussion

This study introduces a novel and highly efficient approach for precise genome editing in CHO-K1 cells, leveraging the TILD-CRISPR method, RNP complexes, and a Ppoly delivery system. Our primary finding demonstrates a significant enhancement in HDR efficiency, achieving a 50% KI rate of the GFP reporter gene, compared to 14% with the commercial Lipofectamine CRISPRMAX™ reagent. This substantial improvement underscores the superior performance of our Ppoly-based delivery system, addressing a critical challenge in CRISPR-mediated gene editing: efficient and safe intracellular delivery.

The observed increase in KI efficiency can be attributed to several key factors. First, the TILD-CRISPR method, which linearizes the donor DNA, likely facilitates more efficient HDR by providing accessible DNA ends for recombination. Recent studies have continued to explore the advantages of linear donor DNA in HDR, demonstrating its superiority over plasmid donors [42,43,44]. Second, the use of preassembled RNP complexes ensures rapid and specific genome editing, minimizing off-target effects and enhancing precision, a crucial aspect highlighted by recent advances in RNP-based CRISPR applications [25,45,46]. Finally—and crucially—the Ppoly delivery system addresses a major bottleneck in CRISPR technology: efficient intracellular delivery. The nanosponge architecture, characterized by its positive surface charge and hydrophobic cavities, facilitates strong complexation with RNP, enhancing cellular uptake and stability. DLS and zeta potential analyses confirmed successful RNP encapsulation and the maintenance of a positive surface charge, which is essential for efficient cellular internalization, consistent with findings from other studies using cationic nanocarriers [47,48].

Furthermore, our study reveals the biocompatibility and low cytotoxicity of the Ppoly delivery system. MTT assays demonstrated cell viability exceeding 80% even at high Ppoly concentrations, indicating minimal adverse effects on cell viability. This is a critical advantage over conventional lipid-based transfection reagents, which often exhibit higher toxicity and elicit immunogenic responses [49]. Recent research has focused on developing biocompatible nanocarriers to overcome these limitations. For instance, studies using biodegradable polymer-based nanoparticles and cyclodextrin-based systems have shown promising results in reducing cytotoxicity and improving gene delivery efficiency [50,51]. Our Ppoly system aligns with this trend, providing a safe and effective platform for CRISPR delivery.

The out-out PCR results revealed a high proportion of homologous insertions (70%) in the Ppoly group, suggesting precise gene integration at the targeted locus. This level of precision is crucial for therapeutic applications, where off-target effects and RI can lead to detrimental outcomes. Conversely, the Lipofectamine CRISPRMAX™ group exhibited a lower KI efficiency and a higher incidence of heterologous insertions, highlighting the limitations of conventional delivery methods. Recent studies have emphasized the importance of minimizing off-target effects in CRISPR-based therapies, with advancements in sgRNA design and Cas protein engineering [52]. Our study contributes to this effort by demonstrating the effectiveness of a delivery system that enhances on-target integration. The gel retardation assays indicated that sgRNA was well encapsulated at all examined concentrations, preventing its migration through the gel. Encapsulation efficiency increased with the RNP:Ppoly ratio up to 1:40, suggesting that Ppoly can effectively encapsulate and deliver CRISPR components. This efficient encapsulation is crucial for ensuring the functionality of RNP complexes within the cellular environment.

While our study demonstrates the significant advantages of the Ppoly delivery system, it is important to acknowledge certain limitations. First, while the 50% KI efficiency is impressive, further optimization may be possible to achieve even higher rates. For example, variations in the Ppoly polymer composition or surface modifications could be explored [33]. Second, although the study focused on GFP reporter gene integration, future research should explore the applicability of this system for delivering larger therapeutic genes and addressing more complex genetic modifications, such as multiple gene insertions or deletions. Third, the long-term stability of the gene insertion should be further analyzed in vivo, and the potential immune response to the nanosponge should be evaluated.

Looking forward, this study opens up several exciting avenues for future research. The Ppoly delivery system could be further optimized by exploring different polymer compositions, sizes, and surface modifications to enhance cellular uptake and target specificity. Additionally, the integration of stimuli-responsive release mechanisms could enable controlled gene editing in response to specific cellular cues, as demonstrated in recent studies using smart nanomaterials [53,54]. The application of this system in various cell types and disease models, including primary cells and induced pluripotent stem cells (iPSCs), could also provide valuable insights into its therapeutic potential. Recent research has focused on using CRISPR-Cas systems in iPSCs for disease modeling and gene therapy [55,56,57]. Moreover, the high efficiency and low toxicity of the Ppoly delivery system make it a promising candidate for gene therapy applications. Future studies should focus on demonstrating the efficacy and safety of this system in preclinical models of genetic diseases, such as cystic fibrosis or muscular dystrophy. Given the versatility of the CRISPR-Cas system, the Ppoly delivery platform can be tailored for a wide range of biotechnological and medical applications, including regenerative medicine, cancer therapy, and drug delivery. For example, recent studies have explored the use of CRISPR-Cas systems for cancer immunotherapy by modifying T cells to target tumor-specific antigens [58,59].

We acknowledge the absence of a non-targeting sgRNA control as a limitation of this study. However, the integration specificity and on-target precision were rigorously validated using out-out PCR and quantitative PCR (qPCR), which are widely recognized as reliable alternative methods for confirming site-specific KI events.

## 4. Materials and Methods

### 4.1. Synthesis of Ppoly

The polymer was synthesized following the procedure described in our previous publication [60]. Briefly, the synthesis was initiated by dissolving 1.00 g (8.81 × 10^−4^ mol) of anhydrous β-Cyclodextrin (βCD) in 7.5 mL of dimethyl sulfoxide (DMSO) at room temperature. Once fully dissolved, 1.11 g (7.93 × 10^−3^ mol) of choline chloride and 1.14 g (7.05 × 10^−3^ mol) of CDI were added sequentially. The resulting transparent solution was subjected to continuous stirring at 90 °C for 120 min, during which an increase in viscosity was observed, indicating successful polymerization. The resultant polymer was subsequently precipitated and washed with acetone, followed by recovery via vacuum filtration. The dried polymer was then dissolved in distilled water and purified through ultrafiltration (cut-off 5 kDa) to remove unreacted reagents, byproducts, and residual solvents. The final product, obtained from the ultrafiltration cell and freeze-dried to yield a white powder, exhibited a mass balance of approximately 85% relative to the theoretical yield, accounting for the combined weights of βCD, choline chloride, and CDI.

### 4.2. DLS and Zeta-Potential Measurement

The particle size using DLS and zeta-potential measurements were performed on a 90-plus particle sizer (Malvern Instrument Ltd., Worcester, UK). RNP–polymer complexes were prepared at varying N/P ratios, defined as the molar ratio of nitrogen atoms in the polymer (from cationic amines) to phosphate groups in the sgRNA. To achieve different N/P ratios (1:1, 1:5, 1:10, 1:20, 1:40), the amount of Ppoly added was adjusted accordingly, while the sgRNA/Cas9 amount was kept constant (240 ng sgRNA and 1250 ng Cas9 in 10 µL). Then, they were left at room temperature for 30 min to form the complexes, after which they were diluted to 1 mL with filtered distilled water. The particle size was measured at 25 °C in a glass cuvette.

### 4.3. FTIR Spectroscopy

Ppoly, RNP, and RNP-loaded Ppoly were analyzed by using FTIR spectroscopic studies (Bruker Optics GmbH, Ettlingen, Germany), in the region of 400–4000 cm^−1^, with a resolution of 4 cm^−1^, to find the existence of interactions between nucleic acids and Ppoly systems. KBr was mixed with the samples (the ratio of KBr to sample is 100:1); then, the KBr pellet was prepared by applying sufficiently high pressure to a homogeneous mixture of KBr and the sample until the pellet turned transparent.

### 4.4. Polymer-sgRNA Complex Formation and Gel Retardation Assay

The complex between Ppoly polymer and sgRNA was made before the transfection experiments. Different *N*/*P* ratios (average number of nitrogen atoms on the cyclodextrin core/number of phosphate groups of sgRNA) of sgRNA/Ppoly complexes were formed (1:1, 1:10, 1:20, 1:40) by mixing 10 µL of sgRNA solution and 10 µL of the polymer solution. To form the complexes, they were mixed, vortexed for 10 s, and incubated at room temperature for 30 min. Finally, the obtained solutions were mixed with loading buffer and loaded onto a 2% agarose gel for gel electrophoresis conducted in 1× TAE buffer (40 mmol/L Tris-acetate and 1 mmol/L EDTA) at 90 V for 45 min. DNA bands were visualized by a Gel Documentation System (Bio-Rad, Hercules, CA, USA). The *N*/*P* ratios were evaluated by using the following formulation:

$$C=\frac{n\times P\times M}{X},n=\frac{N}{P}$$

where *N* was the concentration of nitrogen in cationic compounds (nmol/mL), *P* was the concentration of phosphorus sgRNA (nmol/mL), *X* was the number of nitrogen atoms in cationic compounds, *M* was the molecular weight of cationic compounds (g/mol), and *C* was the concentration of cationic compounds (ng/mL).

### 4.5. Determination of Ppoly/RNP Encapsulation Efficiency

To check the encapsulation efficiency of nanoparticles for RNP, the ultracentrifugation method was used to determine the difference between the total amount of RNP added in the nanoparticle preparation buffer and the amount of free RNP remaining in the aqueous suspension by ultraviolet spectrophotometry at 260 nm. The Ppoly encapsulation efficiency of the process was calculated from equations as indicated below:

$$EE\%=\frac{\left[\left(a-b\right)\times \frac{d}{c}-\left(a-b\right)\right]}{a}\times 100$$

*a*: total amount of RNP, *b*: the free amount of RNP, *c*: nanoparticle weight, and *d*: the total amount of polymer.

### 4.6. Cytotoxicity of Ppoly

The cellular toxicity of the nanocarriers was evaluated using the MTT assay according to the following protocol over a range of concentrations: After culturing and passaging CHO-K1 cells in a T25 flask, the cells were trypsinized and counted. In each well of a 96-well plate, 100 µL of complete culture medium was added, and 10,000 cells were seeded per well. One day later, the cells were ready for the MTT assay. Various concentrations of Ppoly were prepared, added to the cell wells (in triplicate), and incubated for 24 h in a CO_2_ incubator at 37 °C under standard conditions. Three wells were used as controls (untreated CHO-K1 cells) in triplicate. After 24 h, an MTT solution (Thermo Fisher Scientific, Fair Lawn, NJ, USA) with a concentration of 0.5 mg/mL was prepared. The culture medium was removed from the cells, and 100 µL of MTT solution was added. The plate was then incubated for 4 h in a CO2 incubator at 37 °C under standard conditions. After 4 h, the MTT solution was removed, and 100 µL of DMSO was added to each well, which was then incubated for 2 h. Subsequently, the absorbance of the wells was read and analyzed using a microplate reader (Bio-Rad, CA, USA) at 550 and 570 nm.

### 4.7. Design of sgRNAs and Plasmid Construction

The candidate phiC31 pseudo-attP sites are located intergenically on chromosomes 3 (GenBank accession no. APMK01032147.1, nucleotides 1609–3570) and 6 (GenBank accession no. APMK01089843.1, nucleotides 30096–32060) in CHO-K1 cells. The pseudo-attP site on chromosome 6 shares a 58% identity with the wild-type attP [61]. The CRISPOR bioinformatics tool (http://crispor.tefor.net/, accessed on 23 September 2023) [62] was employed to generate sequences for sgRNA targets, selecting high-score sgRNAs based on criteria of high specificity, predicted efficiency, and minimal off-target effects. The sgRNA (5′-AAACAGUUCGAGUACCACAG-3′) targets explicitly the phiC31 pseudo-attP intergenic site on chromosome 6 of the CHO-K1 genome (GenBank accession no. APMK01089843.1 from nucleotide 30096–32060).

### 4.8. Plasmid Donor Linearization

A plasmid donor (GenBank accession no. OQ579020) containing GFP and puromycin expression cassettes (pSV40-GFP-2A-Puro-SV40pA), flanked by 1 kb left and right homology arms corresponding to the Chr6 pseudo-attP site, was used as the template in PCR reactions to create the linearized donor. To prevent double-strand re-cleavage by Cas9 after HDR, the donor plasmid includes two PAM mutations in its homology arms. Plasmids were extracted from transformants selected on LB agar plates with ampicillin. All constructs were validated by gel electrophoresis and purified using an endotoxin-free plasmid extraction kit (Qiagen, Venlo, The Netherlands) following the manufacturer’s instructions.

### 4.9. In Vitro Transcription (IVT)

The target oligonucleotides used to synthesize genome-targeting sgRNA were designed using the GeneArt™ Precision sgRNA Synthesis Kit (Thermo Fisher, Invitrogen™, Waltham, MA, USA). The oligonucleotides were synthesized by GeneScript (Nanjing, China). The subsequent steps of IVT and cleanup were carried out according to the GeneArt™ Precision sgRNA Synthesis Kit protocol.

### 4.10. Cell Culture and Transfection

CHO-K1 cells, obtained from the National Cell Bank of the Pasteur Institute of Iran (NCBI, Tehran, Iran), were cultured in DMEM/F12 medium supplemented with 10% fetal bovine serum (FBS) (Gibco, Grand Island, NE, USA) and 1% penicillin/streptomycin (Gibco, Grand Island, NE, USA). The cells were maintained at 37 °C in a humidified incubator (New Brunswick Scientific, NJ, USA) with 5% CO2 and were passaged into fresh media every three days. A day prior to transfection, CHO cells were seeded at a density of 7 × 10^4^ cells per well in 24-well plates. The puromycin (ABM Good, Richmond, BC, Canada) sensitivity was also determined.

To comparatively assess the efficiency of targeted integration using both the Ppoly delivery system and the commercial CRISPRMAX™ reagent, the media was replaced with penicillin/streptomycin-free media, and then 500 ng of the linearized donor was transfected into CHO-K1 cells using Lipofectamine 3000 reagent (Thermo Fisher, Invitrogen™, Waltham, MA, USA) in accordance with the manufacturer’s protocol prior to delivering the RNPs. The control wells remained untreated.

Five hours post-transfection of the linearized donor, the media in the test wells was replaced with fresh, antibiotic-free media. For the Ppoly delivery method, RNPs were initially assembled in vitro by combining pre-synthesized sgRNA with Cas9 protein (Avan Bio Research, Karaj, Iran) in a 1:1 molar ratio along with the assembly buffer (10 mM Tris-HCl pH 7.5 at 37 °C, 100 mM NaCl, 1 mM EDTA, 1 mM DTT), then incubating the mixture in a 37 °C water bath for 15 min to form the RNP complex. Immediately following synthesis, the RNPs were mixed with the Ppoly nanoparticle at a 1:40 volume ratio, vortexed for 10 s, and incubated at room temperature for 30 min. The resulting mixture was then added to the cells in the test wells.

At the same time, for cell transfection using the commercial CRISPRMAX™ reagent, the RNP complex was prepared as follows: Two 1.5 mL microtubes were prepared. In the first tube, to form the RNP complex, 25 µL of Opti-MEM^®^, 1250 ng of Cas9 protein, 240 ng of sgRNA, and 2.5 µg of Cas9 Plus™ Reagent were added. In the second tube, to dilute the CRISPRMAX™ reagent, 1.5 µL of the reagent was mixed with 25 µL of Opti-MEM^®^. The contents of the first tube were then added to the second tube and incubated at room temperature for 10 min. Finally, the mixture was added to the cells in the test wells.

### 4.11. Puromycin Selection

Seventy-two hours after the initial transfection, the cells were subjected to antibiotic pressure with 6 μg/mL of puromycin (ABM good, BC, Canada). This concentration of the antibiotic was predetermined through MIC testing. The transfected cells using both methods, as well as the control wells, were seeded at approximately 30% confluence in 6-well plates the day before. The culture medium was replaced every two days over a 14-day period until the control cells were eliminated entirely using the puromycin-containing medium.

### 4.12. 5′/3′ Junctions and Out-Out PCR

After the expansion and proliferation of stable, puromycin-resistant clones, genomic DNA was extracted from the cell pool using a genomic DNA extraction kit (Qiagen, Hilden, Germany). The extracted DNA underwent PCR validation to amplify the 5′ and 3′ junctions of the targeted locus and transgene. This was performed using the 2X Taq DNA polymerase master mix (Ampliqon, Odense M, Denmark) following the PCR program: initial denaturation at 95 °C for 5 min, 32 cycles of 95 °C for 30 s, 57 °C for 30 s, and 72 °C for 1 min 30 s (5′ junction) or 1 min 25 s (3′ junction), with a final extension at 72 °C for 10 min. Additionally, following the clonal selection procedure, 5′/3′ junction PCR was performed on the recovered single clones to evaluate the KI efficiency and an out-out PCR was subsequently conducted utilizing genomic DNA of the 5′/3′ Junction PCR-positive clones and primers that target the outer regions of each homology arm to confirm complete transgene integration using the 2X SuperAdd Taq Master mix (Addbio Inc., Daejeon, Republic of Korea). The PCR protocol involved initial denaturation at 95 °C for 5 min, followed by 32 cycles of 95 °C for 30 s, 57 °C for 30 s, and 72 °C for 2 min and 30 s, with a final extension at 72 °C for 5 min. Specific primers, detailed in Supplemental Table S1, were employed in the amplification process. The PCR products were subsequently visualized on a 1% agarose gel.

### 4.13. Limiting Dilution and Single-Cell Cloning

A limiting dilution approach was employed to isolate individual clones from the cell pool. The cells were detached using trypsin and resuspended in an antibiotic-free medium containing 15% FBS. These cells were then plated at a density of one cell per well in 96-well plates. After an incubation period of approximately 14 days, each well containing single-cell clones was transferred to 24-well plates and subsequently expanded to 6-well plates for further analysis.

### 4.14. Evaluation of KI Efficiency

Following the clonal selection phase, genomic DNA was extracted from the recovered clones using the cell lysate protocol [63]. In summary, each clone’s cell pellet was obtained through centrifugation at 1100 rpm for 5 min, then dissolved in 20 μL of 0.2 M NaOH. After a 10-min incubation at 75 °C, the reaction was neutralized by adding 180 μL of 0.04 M Tris-HCl buffer (pH 7.8), yielding the cell lysate. To evaluate and analyze KI efficiency, 2.5 μL of the resulting mixture was used as a template for performing 5′/3′ junction PCRs. The PCR results were then visualized on a 1% agarose gel using the gel documentation system (BioRad Gel Doc XR+ Imaging System, Temecula, CA, USA). The KI efficiency for each group was evaluated by examining single-cell clones obtained from stable cell pools. The efficiency was determined by calculating the percentage of clones positive for 5′/3′ junction PCR. Statistical significance was assessed using a two-sided Fisher’s exact test, with *p*-values less than 0.05 being considered significant.

### 4.15. Copy Number Analysis and Sequencing

The copy number of the inserted GFP reporter gene was determined using relative real-time quantitative reverse transcription PCR (qRT-PCR) with 2X Real-Time PCR Master Green (Ampliqon, Odense M, Denmark) and an ABI 7500 Real-time PCR System (Thermo Fisher, Applied Biosystems™, Waltham, MA, USA). Tests were conducted in duplicate, with a no-template control (NTC) included. Primers targeting the GFP reporter gene and the ACTB (β-actin) reference gene were designed and subsequently validated using melting curve analysis and agarose gel electrophoresis. Amplification was performed on the genomic DNA of the selected positive clones under the following conditions: Initial denaturation at 95 °C for 15 min, followed by 40 cycles of 95 °C for 20 s and 60 °C for 35 s. The copy number was assessed using the cycle threshold (C_t_) and quantified with the 2^−ΔΔCt^ method.

To assess potential mutations within the homology arm junctions and the target locus, the 5′ and 3′ junction PCR amplicons from selected clones were subjected to Sanger sequencing, employing the 5′ junction forward primer and the 3′ junction reverse primer, respectively.

### 4.16. Clonal Stability and Flow Cytometric Analysis

To assess the clonal stability of GFP protein expression over a specified period, three positive clones from each studied group were selected and cultured in a 6-well plate without the antibiotic pressure of puromycin. These clones were passaged and maintained every 3–4 days (twice a week) for a total of 20 passages. A total of 1 × 10^5^ cells from each GFP-expressing clone were harvested and suspended in phosphate-buffered saline (PBS). Mean fluorescent intensity (MFI) and the percentage of GFP-positive cells were evaluated at passages 0, 10, and 20 using a flow cytometer (IndiaMART, Sysmex CyFlow Counter, Jammu Kashmir, India). Notably, non-treated wild-type CHO-K1 was used as a negative control, and the data were analyzed using FlowJo software v10.

### 4.17. Statistical Analysis

All experiments were carried out in triplicate and repeated independently three times. Results are expressed as mean ± SD. Statistical analysis was performed using GraphPad Prism 10.2.3. Differences between groups were evaluated using one-way ANOVA followed by Tukey’s post hoc test for continuous data, and Fisher’s exact test for categorical data. *p* < 0.05 was considered statistically significant.

## 5. Conclusions

In conclusion, the overall efficiency and accuracy of the Ppoly method in achieving successful gene insertion, combined with its reduced cytotoxicity, make it a superior alternative to traditional delivery systems such as Lipofectamine CRISPRMAX™. The innovative approach of utilizing cyclodextrin-based nanosponges for RNP delivery not only enhances genome editing efficiency but also ensures the safety and viability of the target cells. Combining TILD-CRISPR, RNP complexes, and Ppoly offers a versatile and efficient platform for precise genome editing. Our findings underscore the importance of innovative delivery systems in advancing genetic engineering, with significant implications for Biotechnology and Medicine. The superior performance of Ppoly in terms of KI efficiency, encapsulation, and cytotoxicity reduction also makes it a promising tool for future clinical and therapeutic applications.

## Data Availability

The original contributions presented in this study are included in the article. Further inquiries can be directed to the corresponding authors.

## Supplementary Materials

The following supporting information can be downloaded at: https://www.mdpi.com/article/10.3390/ijms262110682/s1.

## Abbreviations

The following abbreviations are used in this manuscript:

CHO	Chinese Hamster Ovary
HDR	Homology-directed repair
NHEJ	Non-homologous end-joining
CD	Cyclodextrin
RNP	Ribonucleoprotein
