# Supplementary material for: Efficient Delivery of CRISPR-Cas9 RNP Complexes with Cyclodextrin-Based Nanosponges for Enhanced Genome Editing: TILD-CRISPR Integration
Source: Int J Mol Sci. 2025 Nov 2;26(21):10682. doi: 10.3390/ijms262110682 (PMC12608112; doi:10.3390/ijms262110682)
Supplement: Supplementary file 1 [file ijms-26-10682-s001.zip › ijms-3900037-supplementary.pdf]

International Journal of
Molecular Sciences

MDPI

Article

Efficient Delivery of CRISPR-Cas9 RNP Complexes with Cyclodextrin-Based Nanosponges for Enhanced Genome Editing: TILD-CRISPR Integration

Shahin Amiri [1,2], Setare Adibzadeh [3], Yusef Khazaei Monfared [4,5], Saeed Kaboli [6], Arash Arashkia [7], Farzaneh Barkhordari [1], Mohammad Mahmoudian [8], Mohammad Hassan Kheirandish [9], Francesco Trotta [5,*] and Fatemeh Davami [1,*]

Supplementary Files

Supplementary Table 1. Primer Sequences Used for 5′/3′ Junction and Out–Out PCR Analyses

Target Region	Primer Name	Sequence (5′→ 3′)
5′ junction	Forward*	TACTGCTTATTGGTTCACTCACC
	Reverse	TCCTCACTACTTCTGGAATAGCTCAG
3′ junction	Forward	CTGCATTCTAGTTGTGGTTTG
	Reverse*	GAGTCTGCTCAGTTTCTTTAAC

* Primers marked with an asterisk were used for out–out PCR validation to confirm complete transgene integration.
